# An empirical research on customer satisfaction study: a consideration of different levels of performance

**DOI:** 10.1186/s40064-016-3208-z

**Published:** 2016-09-15

**Authors:** Yu-Cheng Lee, Yu-Che Wang, Shu-Chiung Lu, Yi-Fang Hsieh, Chih-Hung Chien, Sang-Bing Tsai, Weiwei Dong

**Affiliations:** 1 Department of Technology Management, Chung-Hua University, Hsinchu, 300 Taiwan; 2 Department of Business Administration, Chung-Hua University, Hsinchu, 300 Taiwan; 3 PhD Program of Technology Management, Chung-Hua University, Hsinchu, 300 Taiwan; 4 Department of Food and Beverage Management, Lee-Ming Institute of Technology, New Taipei City, 243 Taiwan; 5 Department of Business Administration, Lee-Ming Institute of Technology, New Taipei City, 243 Taiwan; 6 Department of Food and Beverage Management, Taipei College of Maritime Technology, New Taipei City, 251 Taiwan; 7 Zhongshan Institute, University of Electronic Science and Technology of China, Dongguan, 528402 Guangdong China; 8 School of Economics and Management, Shanghai Maritime University, Shanghai, 201306 China; 9 Law School, Nankai University, Tianjin, 300071 China; 10 School of Business, Dalian University of Technology, Panjin, 124221 China; 11 College of Business Administration, Dongguan University of Technology, Dongguan, 523808 Guangdong China; 12 Department of Psychology, Universidad Santo Tomas de Oriente y Medio Día, Granada, Nicaragua; 13 School of Economics and Management, Shanghai Institute of Technology, Shanghai, 201418 China

**Keywords:** Customer satisfaction, Tourism factory industry, Partial least squares, Business management, Service management

## Abstract

Customer satisfaction is the key factor for successful and depends highly on the behaviors of frontline service providers. Customers should be managed as assets, and that customers vary in their needs, preferences, and buying behavior. This study applied the Taiwan Customer Satisfaction Index model to a tourism factory to analyze customer satisfaction and loyalty. We surveyed 242 customers served by one tourism factory organizations in Taiwan. A partial least squares was performed to analyze and test the theoretical model. The results show that perceived quality had the greatest influence on the customer satisfaction for satisfied and dissatisfied customers. In addition, in terms of customer loyalty, the customer satisfaction is more important than image for satisfied and dissatisfied customers. The contribution of this paper is to propose two satisfaction levels of CSI models for analyzing customer satisfaction and loyalty, thereby helping tourism factory managers improve customer satisfaction effectively. Compared with traditional techniques, we believe that our method is more appropriate for making decisions about allocating resources and for assisting managers in establishing appropriate priorities in customer satisfaction management.

## Background

Traditional manufacturing factories converted for tourism purposes, have become a popular leisure industry in Taiwan. The tourism factories has experienced significant growth in recent years, and more and more tourism factories emphasized service quality improvement, and customized service that contributes to a tourism factory’s image and competitiveness in Taiwan (Wu and Zheng 2014). Therefore, tourism factories has become of greater economic importance in Taiwan. By becoming a tourism factory, companies can establish a connection between consumers and the brand, generate additional income from entrance tickets and on-site sales, and eventually add value to service innovations (Tsai et al. 2012). Because of these incentives, the Taiwanese tourism factory industry has become highly competitive. Customer satisfaction is seen as very important in this case.

Numerous empirical studies have indicated that service quality and customer satisfaction lead to the profitability of a firm (Anderson et al. 1994; Eklof et al. 1999; Ittner and Larcker 1996; Fornell 1992; Anderson and Sullivan 1993; Zeithaml 2000). Anderson and Sullivan (1993) stated that a firm’s future profitability depends on satisfying current customers. Anderson et al. (1994) found a significant relationship between customer satisfaction and return on assets. High quality leads to high levels of customer retention, increase loyalty, and positive word of mouth, which in turn are strongly related to profitability (Reichheld and Sasser 1990). In a tourism factory setting, customer satisfaction is the key factor for successful and depends highly on the behaviors of frontline service providers. Kutner and Cripps (1997) indicated that customers should be managed as assets, and that customers vary in their needs, preferences, buying behavior, and price sensitivity. A tourism factory remains competitive by increasing its service quality relative to that of competitors. Delivering superior customer value and satisfaction is crucial to firm competitiveness (Kotler and Armstrong 1997; Weitz and Jap 1995; Deng et al. 2013). It is crucial to know what customers value most and helps firms allocating resource utilization for continuously improvement based on their needs and wants. The findings of Customer Satisfaction Index (CSI) studies can serve as predictors of a company’s profitability and market value (Anderson et al. 1994; Eklof et al. 1999; Chiu et al. 2011). Such findings provide useful information regarding customer behavior based on a uniform method of customer satisfaction, and offer a unique opportunity to test hypotheses (Anderson et al. 1997).

**Fig. 1:**
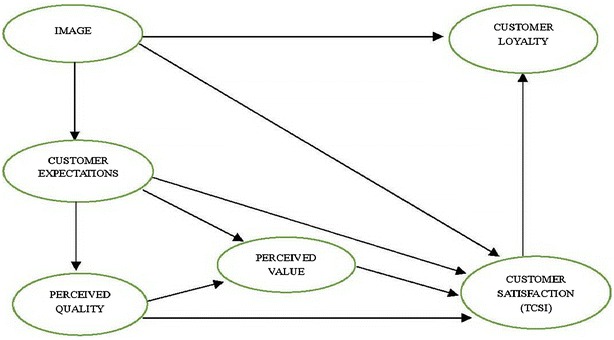
The Taiwan Customer Satisfaction Index model

The basic structure of the CSI model has been developed over a number of years and is based on well-established theories and approaches to consumer behavior, customer satisfaction, and product and service quality in the fields of brands, trade, industry, and business (Fornell 1992; Fornell et al. 1996). In addition, the CSI model leads to superior reliability and validity for interpreting repurchase behavior according to customer satisfaction changes (Fornell 1992). These CSIs are fundamentally similar in measurement model (i.e. causal model), they have some obvious distinctions in model’s structure and variable’s selection. Take full advantages of other nations’ experiences can establish the Taiwan CSI Model which is suited for Taiwan’s characters. Thus, the ACSI and ECSI have been used as a foundation for developing the Taiwan Customer Satisfaction Index (TCSI). The TCSI was developed by Chung Hua University and the Chinese Society for Quality in Taiwan. The TCSI provides Taiwan with a fair and objective index for producing vital information that can help the country, industries, and companies improve competitiveness. Every aspect of the TCSI that influences overall customer satisfaction can be measured through surveys, and every construct has a cause–effect relationship with the other five constructs (Fig. 1). The relationships among the different aspects of the TCSI are different from those of the ACSI, but are the same as those of the ECSI (Lee et al. 2005, 2006).

The traditional CSI model for measuring customer satisfaction and loyalty is restricted and does not consider the performance of firms. Moreover, as theoretical and empirical research has shown, the relationship between attribute-level performance and overall satisfaction is asymmetric. If the asymmetries are not considered, the impact of the different attributes on overall satisfaction is not correctly evaluated (Anderson and Mittal 2000; Matzler and Sauerwein 2002; Mittal et al. 1998; Matzler et al. 2003, 2004). Few studies have investigated CSI models that contain different levels of performance (satisfaction), especially in relation to satisfaction levels of a tourism factory. To evaluate overall satisfaction accurately, the impact of the different levels of performance should be considered (Matzler et al. 2004). The purpose of this study is to apply the TCSI model that contains different levels of performance to improve and ensure the understanding of firm operational efficiency by managers in the tourism factory. A partial least squares (PLS) was performed to test the theoretical model due to having been successfully applied to customer satisfaction analysis. The PLS is well suited for predictive applications (Barclay et al. 1995) and using path coefficients that regard the reasons for customer satisfaction or dissatisfaction and providing latent variable scores that could be used to report customer satisfaction scores. Our findings provide support for the application of TCSI model to derive tourist satisfaction information.

## Literature review

### National customer satisfaction index (CSI)

The CSI model includes a structural equation with estimated parameters of hidden categories and category relationships. The CSI can clearly define the relationships between different categories and provide predictions. The basic CSI model is a structural equation model with latent variables which are calculated as weighted averages of their measurement variables, and the PLS estimation method calculates the weights and provide maximum predictive power of the ultimate dependent variable (Kristensen et al. 2001). Many scholars have identified the characteristics of the CSI (Karatepe et al. 2005; Malhotra et al. 1994).

Although the core of the models are in most respects standard, they have some obvious distinctions in model’s structure and variable’s selection so that their results cannot be compared with each other and some variations between the SCSB (Swedish), the ACSI (American), the ECSI (European), the NCSB (Norwegian) and other indices. For example, the image factor is not employed in the ACSI model (Johnson et al. 2001); the NCSB eliminated customer expectation and replaced with corporate image; the ECSI model does not include the customer complaint as a consequence of satisfaction. Many scholars have identified the characteristics of the CSI (Karatepe et al. 2005; Malhotra et al. 1994). The ECSI model distinguishes service quality from product quality (Kristensen et al. 2001) and the NCSB model applies SERVQUAL instrument to evaluate service quality (Johnson et al. 2001). A quality measure of a single customer satisfaction index is typically developed according to a certain type of culture or the culture of a certain country. When developing a system for measuring or evaluating a certain country or district’s customer satisfaction level, a specialized customer satisfaction index should be developed.

As such, the ACSI and ECSI were used as a foundation to develop the TCSI. The TCSI was developed by Chung Hua University and the Chinese Society for Quality. Every aspect of the TCSI that influences overall customer satisfaction can be measured through surveys, and every construct has a cause–effect relationship with the other five constructs. The TCSI assumes that currently: (1) Taiwan corporations have ability of dealing with customer complaints; customer complaints have already changed from a factor that influences customer satisfaction results to a factor that affects quality perception; (2) The expectations, satisfaction and loyalty of customers are affected by the image of the corporation. The concept that customer complaints are not calculated into the TCSI model is that they were removed based on the ECSI model (Lee et al. 2005, 2006, 2014a, b; Guo and Tsai 2015; Tsai et al. 2015a, b; 2016a).

### TCSI model and service quality

Service quality is frequently used by both researchers and practitioners to evaluate customer satisfaction. It is generally accepted that customer satisfaction depends on the quality of the product or service offered (Anderson and Sullivan 1993). Numerous researchers have emphasized the importance of service quality perceptions and their relationship with customer satisfaction by applying the NCSI model (e.g., Ryzin et al. 2004; Hsu 2008; Yazdanpanah et al. 2013; Chiu et al. 2011; Temizer and Turkyilmaz 2012; Mutua et al. 2012; Dutta and Singh 2014). Ryzin et al. (2004) applied the ACSI to U.S. local government services and indicated that the perceived quality of public schools, police, road conditions, and subway service were the most salient drivers of satisfaction, but that the significance of each service varied among income, race, and geography. Hsu (2008) proposed an index for online customer satisfaction based on the ACSI and found that e-service quality was more determinative than other factors (e.g., trust and perceived value) for customer satisfaction. To deliver superior service quality, an online business must first understand how customers perceive and evaluate its service quality. This study developed a basic model for using the TCSI to analyze Taiwan’s tourism factory services. The theoretical model comprised 14 observation variables and the following six constructs: image, customer expectations, perceived quality, perceived value, customer satisfaction, and loyalty.

## Methods

### Research methods

The measurement scale items for this study were primarily designed using the questionnaire from the TCSI model. In designing the questionnaire, a 10-point Likert scale (with anchors ranging from strongly disagree to strongly agree) was used to reduce the statistical problem of extreme skewness (Fornell et al. 1996; Qu et al. 2015; Tsai 2016; Tsai et al. 2016b; Zhou et al. 2016). A total of 14 items, organized into six constructs, were included in the questionnaire. The primary questionnaire was pretested on 30 customers who had visited a tourism factory. Because the TCSI model is preliminary research in the tourism factory, this study convened a focus group to decide final attributes of model. The focus group was composed of one manager of tourism factory, one professor in Hospitality Management, and two customers with experience of tourism factory.

We used the TCSI model (Fig. 1) to structure our research. From this structure and the basic theories of the ACSI and ECSI, we established the following hypotheses:

#### **H1**

Image has a strong influence on tourist expectations.

#### **H2**

Image has a strong influence on tourist satisfaction.

#### **H3**

Image has a strong influence on tourist loyalty.

#### **H4**

Tourist expectations have a strong influence on perceived quality.

#### **H5**

Tourist expectations have a strong influence on perceived values.

#### **H6**

Tourist expectations have a strong influence on tourist satisfaction.

#### **H7**

Perceived quality has a strong influence on perceived value.

#### **H8**

Perceived quality has a strong influence on tourist satisfaction.

#### **H9**

Perceived value has a strong influence on tourist satisfaction.

#### **H10**

Customer satisfaction has a strong influence on tourist loyalty.

The content of our surveys were separated into two parts; customer satisfaction and personal information. The definitions and processing of above categories are listed below:

1. Part 1 of the survey assessed customer satisfaction by measuring customer levels of tourism factory image, expectations, quality perceptions, value perceptions, satisfaction, and loyalty toward their experience, and used these constructs to indirectly survey the customer’s overall evaluation of the services provided by the tourism factory.
2. Part 2 of the survey collected personal information: gender, age, family situation, education, income, profession, and residence.

The six constructs are defined as follows:

1. Image reflects the levels of overall impression of the tourism factory as measured by two items: (1) word-of-mouth reputation, (2) responsibility toward concerned parties that the tourist had toward the tourism factory before traveling.
2. Customer expectations refer to the levels of overall expectations as measured by two items: (1) expectations regarding the service of employees, (2) expectations regarding reliability that the tourist had before the experience at the tourism factory.
3. Perceived quality was measured using three survey measures: (1) the overall evaluation, (2) perceptions of reliability, (3) perceptions of customization that the tourist had after the experience at the tourism factory.
4. Perceived value was measured using two items: (1) the cost in terms of money and time (2) a comparison with other tourism factories.
5. Customer satisfaction represents the levels of overall satisfaction was captured by two items: (1) meeting of expectations, (2) closeness to the ideal tourism factory.
6. Loyalty was measured using three survey measures: (1) the probabilities of visiting the tourism factory again (2) attending another activity held by the tourism factory, (3) recommending the tourism factory to others.

### Data collection and analysis

The survey sites selected for this study was the parking lots of one food tourism factory in Taipei, Taiwan. A domestic group package and individual tourists were a major source of respondents who were willing to participate in the survey and completed the questionnaires themselves based on their perceptions of their factory tour experience. Four research assistants were trained to conduct the survey regarding to questionnaire distribution and sampling.

To minimize prospective biases of visiting patterns, the survey was conducted at different times of day and days of week—Tuesday, Thursday, Saturday for the first week; Monday, Wednesday, Friday and Sunday for the next week. The afternoon time period was used first then the morning time period in the following weeks. The data were collected over 1 month period.

Of 300 tourists invited to complete the questionnaire, 242 effective responses were obtained (usable response rate of 80.6 %). The sample of tourists contained more females (55.7 %) than males (44.35 %). More than half of the respondents had a college degree or higher, 28 % were students, and 36.8 % had an annual household income of US $10,000–$20,000. The majority of the respondents (63.7 %) were aged 20–40 years.

## Results

### Comparison of the TCSI models for satisfied and dissatisfied customers

Researchers have claimed that satisfaction levels differ according to gender, age, socioeconomic status, and residence (Bryant and Cha 1996). Moreover, the needs, preferences, buying behavior, and price sensitivity of customers vary (Kutner and Cripps 1997). Previous studies have demonstrated that it is crucial to measure the relative impact of each attribute for high and low performance (satisfaction) (Matzler et al. 2003, 2004). To determine the reasons for differences, a satisfaction scale was used to group the sample into *satisfied* (8–10) and *dissatisfied* (1–7) customers.

**Fig. 2:**
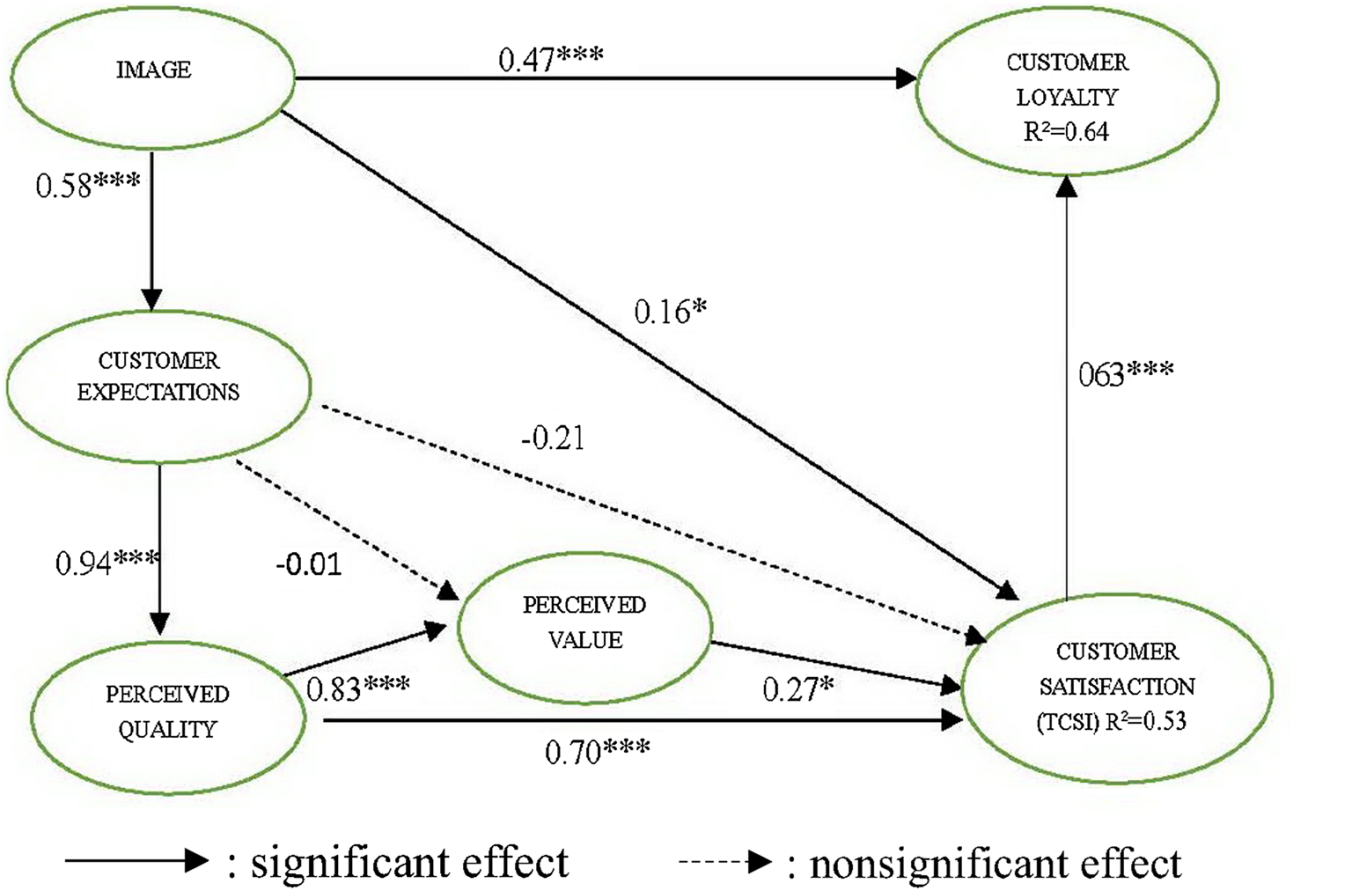
Path estimate of the TCSI model for satisfied customers. *p < 0.05; **p < 0.01; ***p < 0.001

**Fig. 3:**
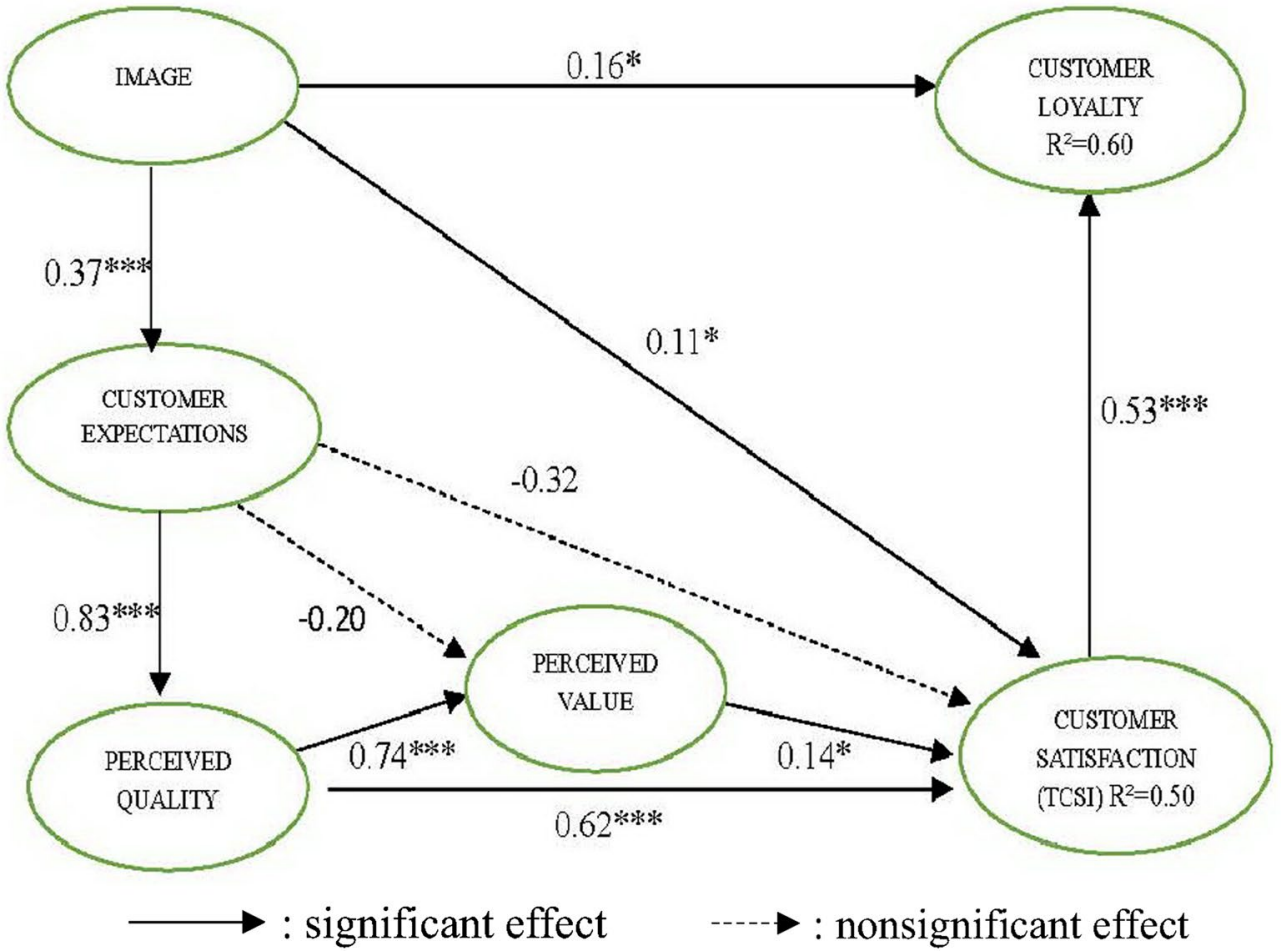
Path estimate of the TCSI model for dissatisfied customers. *p < 0.05; **p < 0.01; ***p < 0.001

The research model was tested using SmartPLS 3.0 software, which is suited for highly complex predictive models (Wold 1985; Barclay et al. 1995). In particular, it has been successfully applied to customer satisfaction analysis. The PLS method is a useful tool for obtaining indicator weights and predicting latent variables and includes estimating path coefficients and R^2^ values. The path coefficients indicate the strengths of the relationships between the dependent and independent variables, and the R^2^ values represent the amount of variance explained by the independent variables. Using Smart PLS, we determined the path coefficients. Figures 2 and 3 show ten path estimates corresponding to the ten research hypothesis of TCSI model for satisfied and dissatisfied customers. Every path coefficient was obtained by bootstrapping the computation of R^2^ and performing a *t* test for each hypothesis. Fornell et al. (1996) demonstrated that the ability to explain the influential latent variables in a model is an indicator of model performance, in particular the customer satisfaction and customer loyalty variables. From the results shown, the R^2^ values for the customer satisfaction were 0.53 vs. 0.50, respectively; and the R^2^ value for customer loyalty were 0.64 vs. 0.60, respectively. Thus, the TCSI model explained 53 vs. 50 % of the variance in customer satisfaction; 64 vs. 60 % of that in customer loyalty as well.

According to the path coefficients shown in Figs. 2 and 3, image positively affected customer expectations (β = 0.58 vs. 0.37), the customer satisfaction (β = 0.16 vs. 0.11), and customer loyalty (β = 0.47 vs. 0.16). Therefore, H1–H3 were accepted. Customer expectations were significantly related to perceived quality (β = 0.94 vs. 0.83). However, customer expectations were not significantly related to perceived value shown as dotted line (β = −0.01 vs. −0.20) or the customer satisfaction, shown as dotted line (β = −0.21 vs. −0.32). Thus, H4 was accepted but H5 and H6 were not accepted. Perceived value positively affected the customer satisfaction (β = 0.27 vs. 0.14), supporting H7. Accordingly, the analysis showed that each of the antecedent constructs had a reasonable power to explain the overall customer satisfaction. Furthermore, perceived quality positively affected the customer satisfaction (β = 0.70 vs. 0.62), as did perceived value (β = 0.83 vs. 0.74). These results confirm H8 and H9. The path coefficient between the customer satisfaction and customer loyalty was positive and significant (β = 0.63 vs. 0.53). This study tested the suitability of two TCSI models by analyzing the tourism factories in Taiwan. The results showed that the TCSI models were all close fit for this type of research. This study provides empirical evidence of the causal relationships among perceived quality, image, perceived value, perceived expectations, customer satisfaction, and customer loyalty.

To observe the effects of antecedent constructs of perceived value (e.g., customer expectation and perceived quality), customer expectations were not significantly related to perceived value for either satisfied or dissatisfied customers. Furthermore, satisfied customers were affected more by perceived quality (β = 0.83 vs. 0.74), as shown in Table 1. Regarding the effect of the antecedents of customer satisfaction (e.g., image, customer expectations, perceived value and perceived quality), the total effects of perceived quality on the customer satisfaction of satisfied and dissatisfied customers were 0.92 and 0.72. The total effects of image on the customer satisfaction of satisfied and dissatisfied customers were 0.45 and 0.19. Thus, the satisfaction level of satisfied customers was affected more by perceived quality. Consequently, regarding customer satisfaction, perceived quality is more important than image for satisfied and dissatisfied customers. Numerous researchers have emphasized the importance of service quality perceptions and their relationship with customer satisfaction by applying the CSI model (e.g., Ryzin et al. 2004; Hsu 2008; Yazdanpanah et al. 2013; Chiu et al. 2011; Temizer and Turkyilmaz 2012; Mutua et al. 2012; Dutta and Singh 2014). This is consistent with the results of previous research ( O’Loughlin and Coenders 2002; Yazdanpanah et al. 2013; Chiu et al. 2011; Chin and Liu 2015; Chin et al. 2016).

**Table 1:** Path estimates of the satisfied and dissatisfied customer CSI model

Path	Effected sign	Path estimate	
		Satisfied	Dissatisfied
Expectation → value	−	−0.009	−0.203
Quality → value	+	0.83***	0.74***
Image → CS	+	0.16*	0.11*
Expectation → CS	−	−0.21	−0.32
Value → CS	+	0.27*	0.14*
Quality → CS	+	0.80***	0.62***
Image → expectation	+	0.58***	0.37***
Expectation → Quality	+	0.94***	0.73***
Image → loyalty	+	0.47***	0.16*
CS → loyalty	+	0.63***	0.14*

*CS* customer satisfaction

* p < 0.05; ** p < 0.01; *** p < 0.001

With respect to the effect of the antecedents of customer loyalty (e.g., image and customer satisfaction), the total effects of image on customer loyalty for satisfied and dissatisfied customers were 0.57 and 0.21. In other words, the customer loyalty of satisfied customers was affected more by customer satisfaction. Customer satisfaction was significantly related to the customer loyalty of both satisfied and dissatisfied customers, and satisfied customers were affected more by customer satisfaction (*β* = 0.63 vs. 0.14). Consequently, regarding customer loyalty, customer satisfaction is more important than image for both satisfied and dissatisfied customers. Numerous studies have shown that customer satisfaction is a crucial factor for ensuring customer loyalty (Barsky 1992; Smith and Bolton 1998; Hallowell 1996; Grønholdt et al. 2000). This study empirically supports the notion that customer satisfaction is positively related to customer loyalty.

The TCSI model has a predictive capability that can help tourism factory managers improve customer satisfaction based on different performance levels. Our model enables managers to determine the specific factors that significantly affect overall customer satisfaction and loyalty within a tourism factory. This study also helps managers to address different customer segments (e.g., satisfied vs. dissatisfied); because the purchase behaviors of customers differ, they must be treated differently. The contribution of this paper is to propose two satisfaction levels of CSI models for analyzing customer satisfaction and loyalty, thereby helping tourism factory managers improve customer satisfaction effectively.

Fornell et al. (1996) demonstrated that the ability to explain influential latent variables in a model, particularly customer satisfaction and customer loyalty variables, is an indicator of model performance. However, the results of this study indicate that customer expectations were not significantly related to perceived value for either satisfied or dissatisfied customers. Moreover, they were affected more by perceived quality of customer satisfaction. Numerous researchers have found that the construct of customer expectations used in the ACSI model does not significantly affect the level of customer satisfaction (Johnson et al. 1996, 2001; Martensen et al. 2000; Anderson and Sullivan 1993).

Through the overall effects, this study derived several theoretical findings. First, the factors with the largest influence on customer satisfaction were perceived quality and perceived expectations, despite the results showing that customer expectations were not significantly related to perceived value or customer satisfaction. Hence, customer expectations indirectly affected customer satisfaction through perceived quality. Accordingly, perceived quality had the greatest influence on customer satisfaction. Likewise, our results also show that satisfied customers were affected more by perceived quality than dissatisfied customers. This study determined that perceived quality, whether directly or indirectly, positively influenced customer satisfaction. This result is consistent with those of Cronin and Taylor (1992), Cronin et al. (2000), Hsu (2008), Ladhari (2009), Terblanche and Boshoff (2010), Deng et al. (2013), and Yazdanpanah et al. (2013).

Second, the factors with the most influence on customer loyalty were image and customer satisfaction. The results of this study demonstrate that the customer loyalty of satisfied customers was affected more by customer satisfaction. Consequently, regarding customer loyalty, customer satisfaction is more important than image for satisfied customers. Lee (2015) found that higher overall satisfaction increased the possibility that visitors will recommend and reattend tourism factory activities. Moreover, numerous studies have shown that customer satisfaction is a crucial factor for ensuring customer loyalty (Barsky 1992; Smith and Bolton 1998; Hallowell 1996; Su 2004; Deng et al. 2013). In initial experiments on ECSI, corporate image was assumed to have direct influences on customer expectation, satisfaction, and loyalty. Subsequent experiments in Denmark proved that image affected only expectation and satisfaction and had no relationship with loyalty (Martensen et al. 2000). In early attempts to build the ECSI model, image was defined as a variable involving not only a company’s overall image but products or brand awareness; thus image is readily connected with customer expectation and perception. Therefore, this study contributes to relevant research by providing empirical support for the notion that customer satisfaction is positively related to customer loyalty.

In addition to theoretical implications, this study has several managerial implications. First, the TCSI model has a satisfactory predictive capability that can help tourism factory managers to examine customer satisfaction more closely and to understand explicit influences on customer satisfaction for different customer segments by assessing the accurate causal relationships involved. In contrast to general customer satisfaction surveys, the TCSI model cannot obtain information on post-purchase customer behavior to improve customer satisfaction and achieve competitive advantage.

Second, this study not only indicated that each of the antecedent constructs had reasonable power to explain customer satisfaction and loyalty but also showed that perceived quality exerts the largest influence on the customer satisfaction of Taiwan’s tourism factory industry. Therefore, continually, Taiwan’s tourism factories must endeavor to enhance their customer satisfaction, ideally by improving service quality. Managers of Taiwan’s tourism factories must ensure that service providers deliver consistently high service quality.

Third, this research determined that the factors having the most influence on customer loyalty were image and customer satisfaction. Therefore, managers of Taiwan’s tourism factories should allow customer expectations to be fulfilled through experiences, thereby raising their overall level of satisfaction. Regarding image, which refers to a brand name and its related associations, when tourists regard a tourism factory as having a positive image, they tend to perceive higher value of its products and services. This leads to a higher level of customer satisfaction and increased chances of customers’ reattending tourism factory activities.

## Conclusion

Different performance levels exist in how tourists express their opinions about various aspects of service quality and satisfaction with tourism factories. Customer segments can have different preferences depending on their needs and purchase behavior. Our findings indicate that tourists belonging to different customer segments (e.g., satisfied vs. dissatisfied) expressed differences toward service quality and customer satisfaction. Thus, the management of Taiwan’s tourism factories must notice the needs of different market segments to meet their individual expectations. This study proposes two satisfaction levels of CSI models for analyzing customer satisfaction and loyalty, thereby helping tourism factory managers improve customer satisfaction effectively. Compared with traditional techniques, we believe that our method is more appropriate for making decisions about allocating resources and for assisting managers in establishing appropriate priorities in customer satisfaction management.

### Limitations and suggestions for future research

This study has some limitations. First, the tourism factory surveyed in this study was a food tourism factory operating in Taipei, Taiwan, and the present findings cannot be generalized to the all tourism factory industries. Second, the sample size was quite small for tourists (N = 242). Future research should collect a greater number of samples and include a more diverse range of tourists. Third, this study was preliminary research on tourism factories, and domestic group package tourists were a major source of the respondents. Future studies should collect data from international tourists as well.
